# Prevalence and associated factors of obsessive-compulsive disorder among the general population of Latvia

**DOI:** 10.1192/j.eurpsy.2023.534

**Published:** 2023-07-19

**Authors:** V. Vinogradova, A. Kivite-Urtane, J. Vrublevska, E. Rancans

**Affiliations:** ^1^Department of Psychiatry and Narcology; ^2^Department of Public Health and Epidemiology; ^3^Institute of Public Health, Riga Stradins University, Riga, Latvia

## Abstract

**Introduction:**

Obsessive-compulsive disorder (OCD) is one of the most severe and potentially disabling disorders among all anxiety disorders (Hendriks *et al.* J Affect Disord 2014; 166:227-33). There is no available information about the prevalence of OCD in the general population of Latvia.

**Objectives:**

The aim of our study was to assess the one-month prevalence of OCD in the general population of Latvia and determine the associated factors.

**Methods:**

The study was conducted on a representative sample of the Latvian adult population (n=2687), selected using a stratified random sampling method. Computer assisted face-to-face interviews were carried out between November 2019 and March 2020 in the households of the respondents. The OCD, and possible comorbid diagnoses, were assessed using the MINI International Neuropsychiatric Interview (M.I.N.I.). Anxiety symptoms were assessed with 7-item Generalized Anxiety Disorder (GAD-7) scale: a score of ≥5 indicated the presence of at least mild symptoms of anxiety. Patient-Health Questionnaire-9 (PHQ-9) was used for assessing comorbid depressive symptoms and a score of ≥10 indicated the presence of clinically relevant depressive symptoms. Descriptive statistics and binary logistic regression were applied.

**Results:**

In total 1238 males (46.1%) and 1449 females (53.9%) were recruited. Detected one-month prevalence of OCD was 0.6% (n=16). After adjustment by all analysed factors (n=13) simultaneously, the odds ratio of having OCD adjusted for confounders (aOR) was higher in respondents younger than 44 y.o (vs. >44, aOR 14.2, p=0.007): 81.3% of all respondents with diagnosed OCD were younger than 44 y.o.; The odds were statistically significantly higher in respondents with diagnosed severe anxiety (vs. no anxiety, aOR 26.0, p<0.001), alcohol use disorder (vs. no disorder, aOR 7.9, p=0.004) and suicidal behaviour disorder (vs. no suicidality, aOR 5.3, p=0.01).

**Conclusions:**

One-month prevalence of OCD in Latvian general adult population is 0.6%. Young age, diagnosed severe anxiety, suicidal behaviour and alcohol use disorder are significantly associated with the OCD.

**Disclosure of Interest:**

None Declared

